# Incidence of hospitalization and mortality due to stroke in young adults, residents of developed regions in Brazil, 2008-2018

**DOI:** 10.1371/journal.pone.0242248

**Published:** 2020-11-16

**Authors:** Henrique de Moraes Bernal, Luiz Carlos de Abreu, Italla Maria Pinheiro Bezerra, Fernando Adami, Jessica Miwa Takasu, João Victor Ji Young Suh, Silmara de Lira Ribeiro, Edige Felipe de Sousa Santos

**Affiliations:** 1 Laboratório de Delineamento em Estudos e Escrita Científica, Centro Universitário de Saúde do ABC, Santo André, São Paulo, Brazil; 2 Programa de Mestrado em Políticas Públicas e Desenvolvimento Local, Escola Superior de Ciências da Santa Casa de Misericórdia, Vitória, Brazil; 3 Graduate Entry Medical School, University of Limerick, Limerick, Ireland; 4 Faculdade de Saúde Pública, Universidade de São Paulo (USP), São Paulo, São Paulo, Brazil; University of Tasmania, AUSTRALIA

## Abstract

**Introduction:**

We evaluated trends in hospitalization incidence and mortality due to hemorrhagic and ischemic stroke in young adults, according to gender and developed regions in Brazil.

**Methods:**

Between 2008–2018, we performed a population-based time-series study using official hospitalization and death data due to stroke, in individuals aged 10–49 years, from Southeast and South, Brazil. Data were based on reports from the Unified Health System of Hospital Information System and Mortality Information System. Stroke was defined by the International Classification of Diseases, 10^th^ revision (I60–I63). A Prais-Winsten regression model was performed and the Annual Percentage Change was calculated.

**Results:**

In total, 78,123 hospitalizations of individuals aged 10–49 years were recorded, of which 59,448 (76%) resulted from hemorrhagic stroke (HS). The hospitalizations for HS was significantly decreased (- 4.37%) among men and women in both regions. The hospitalizations for ischemic stroke (IS) was flat, except between 2011 and 2018, when IS hospitalization rates increased. In the analysis by states, HS hospitalizations declined across all states, except for Espírito Santo, where it remained unchanged (p > 0.05). IS flat hospitalizations were observed in all states, except Espírito Santo, where it increased by 24.93%. In terms of mortality, 28,625 deaths were recorded, of which 26,548 (92.7%) resulted from HS. HS mortality decreased significantly by -3.48%and IS mortality by -3.84%. Decreases also occurred in all Southeast and South states (p < 0.05). IS remained unchanged across all states, except Minas Gerais, where it decreased by -14.95%.

**Conclusions:**

We identified a decline in the hospitalizations and mortality of HS and a flat trend for IS in developed regions of Brazil. The recent period (2011–2018) demonstrated increasing rates in the hospitalizations of IS in both regions and genders. The mortality rates for HS and IS decreased between 2008–2018 in Southeast and South Brazil for both genders.

## Introduction

Stroke is the leading cause of death in Brazil [1], hospitalization in Brazilian Unified Health System [2] and disability [3], especially in adults of productive age [4].

It is classified into two types: ischemic stroke (IS) and hemorrhagic stroke (HS). In young populations, stroke is not considered a rare event; previous studies have demonstrated heterogeneous results on this issue, with standardized rates ranging from 8.70–21.02 [5].

According to one Brazilian study in young adults, stroke mortality began to decrease in the 1980s onwards [6], while there was a 62% increase (incidence rate ratios, 1.62; CI 95% [1.10: 2.40]) in the disease incidences between 2005 and 2015 [7]. Stroke is often disabling; therefore, this trend poses huge threats to socioeconomic stability, especially in developing countries [8].

Despite declining stroke mortality rates in Brazil [6], the disease is a serious concern, especially when considering other socioeconomic consequences of Cerebrovascular Diseases, such as disability, high social costs [9] and severe disability in approximately 66% of survivors [10]. Also, the disease burden is geographically unequal, which 85% of stroke deaths concentrated in developing countries, such as Brazil [11].

Although stroke occurrence increases considerably with age, in Brazil, there is limited information on population trends in hospitalization and mortality rates in young adults, in terms of type of stroke, gender, age [12] across regions/states.

Within this limited literature, a study conducted between 2008 and 2012 did not discriminate trends according to gender or disease types [1]: these authors investigated stroke but without discriminating the category [1], whereas in another study, authors only stratified stroke levels in a specific Brazilian population municipality [7]. Therefore, the literature does not contain information specifically of a Brazilian context that demonstrates contemporary disease incidence of hospitalizations and mortality rates in the country.

To address this lack of information, our study evaluated incidence trends in hospitalization and mortality by stroke type, in young adults in two developed regions of Brazil, (Southeast and South), according to gender (male and female), between 2008 and 2018.

## Materials and methods

This was an ecological time-series study, using public and official stroke hospitalization and death data extracted from the Information System on Mortality (SIM) and the Unified Health System Hospital Information System (SIH/SUS). Both platforms contain information available on the Unified Health System Department of Informatics (DATASUS—Departamento de Informática do Sistema Único de Saúde, https://datasus.saude.gov.br/), maintained by the Brazilian Ministry of Health. All data were fully anonymized before we accessed them. The study was approved by the Human Research Ethics Committee of the Centro Universitário Saúde ABC (process number 214.586).

### Study population, cause of death and hospital admissions

Data were collected by residential place of hospitalization and death. We selected highly urbanized Southeast and South Brazilian regions (i.e., developed regions), with young population estimated at 53,075,661 (20.69%) million and 17,846,228 (6.99%), respectively [13]. Individuals aged 10–49 years-old were assessed.

Southeast Brazil comprises four states: São Paulo, Rio de Janeiro, Minas Gerais and Espírito Santo. South Brazil comprises three states: Santa Catarina, Paraná and Rio Grande do Sul. Collected hospital admission data corresponded to 2008–2018. Collected death data corresponded to 2008–2017. Population data were extracted from the Brazilian Institute of Geography and Statistics (IBGE—Instituto Brasileiro de Geografia e Estatística, www.ibge.gov.br), which originated from the 2010 census, and its projections for other years in between.

Stroke was classified according to the 10^th^ International Classification of Diseases (ICD-10), for non-traumatic subarachnoid hemorrhage (I60), non-traumatic subarachnoid hemorrhage (I61), other and unspecified non-traumatic subarachnoid hemorrhage (I62) and cerebral infarctions (I63). Subjects recruited were aged 10–49 years-old, and were subdivided into age groups (10–14; 15–19; 20–24; 25–29; 30–34; 35–39; 40–44 and 45–49 years-old), defined as young adults [14]. Calendar years corresponded to the years between 2008 and 2018, available on the DATASUS database.

### Information system

SIH/SUS and SIM receives, processes, verifies, validates, and provides information on hospitalizations (SIH/SUS) and deaths (SIM) from people who use one of the health care facilities registered in the Unified Health System (SUS), which comprises 92.3% [15] of health care in Brazil. Hospitalization and death data are available on the Department of Informatics of the National Health System (DATASUS) website, the country’s official, free and public health information database. This was extracted to collect hospitalization and death data from stroke. These platforms are used for the development of public health policies in Brazil.

### Study variables and data extraction

Hospitalization incidence and mortality rates were constructed according to 1) gender (male and female); 2) age group (10–14, 15–19, 20–24, 25–29, 30–34, 35–39, 40–44 and 45–49 years); 3) disease types (hemorrhagic and ischemic); 4) year (2008–2018); and 5) Regions (Southeast and South) and States (São Paulo, Rio de Janeiro, Minas Gerais, Espírito Santo, Rio Grande do Sul, Paraná e Santa Catarina). Rates were expressed per 100,000 inhabitants in a standardized procedure, considering as standard the percentage distribution of the world’s population provided by the World Health Organization (year 2000–2025) for each age group [16]. The TABNET and TABWIN software were used to analyze the data. These tools were developed by DATASUS for quick tabbing on.DBF files. Data were collected by two independent researchers to identify discrepancies.

### Statistical analyzes

Population hospitalization and mortality rates were calculated, stratified by gender, age group, state and year, and expressed per 100,000 inhabitants. Analyzes were performed for both stroke type groups (I60–I63), and also separately by hemorrhagic types (I60–I62), and ischemic type (I63) [1, 17]. This procedure ensured a sufficient number of cases and stability for analyzes.

For trend analyzes, we used methodological indications, formulated by Antunes et al. (2015) [18]. Time-series construction rates were calculated using a Prais-Winsten regression model, which allowed first-order autocorrelation corrections to be performed on values, organized by time. Thus, the following values were estimated: angular coefficient (β) and respective probability (p), considering a significance level of 95% (confidence interval (CI): 95%).

The modelling process included transforming rates into a logarithmic base function 10. Furthermore, the Durbin-Watson test was also used, and growth or decline rates were calculated according to Annual Percent Change (APC) values, specific to gender, age and stroke type, according to developed regions/states. This procedure facilitated the classification of temporal trends rates as increasing, decreasing or flat, and quantified the annual increment rate.

Moreover, to facilitate visualization of trends and to reduce white noise in the historical series graphs, a third order centered moving averages technique was performed for trends. All analyzes were conducted using the Stata 15.1 statistical program (College Station, TX, USA. 2018).

## Results

### Hospitalization incidences

In total, 78,123 hospitalizations due to stroke were recorded, of which 59,448 (76%) resulted from HS. Hospitalization incidence rates were higher in South, when compared to Southeast Brazil. In terms of gender stratification, the incidence of annual hospitalization (x 100,000 persons) for men was 8.25 in Southeast Brazil, and 12.29 in South Brazil. The incidence of annual hospitalization (x 100,000 persons) for women was 8.32 in Southeast Brazil, and 11.53 in South Brazil (Table 1).

**Table 1:** Hospitalization and mortality rates due to stroke (x 100,000 inhabitants) in young adults, according to demographic and clinical characteristics. Southeast and South regions, Brazil 2008–2018.

Demographic and clinical characteristics	Hospitalizations (n)	Annual incidence (x 100,000) **Southeast**	Annual incidence (x 100,000) **South**
**Gender**			
Male	39,548	8.78	13.20
Female	38,575	8.58	12.02
**Age range** (years)			
10–14	1,562	1.43	0.64
15–19	3,141	2.66	1.34
20–24	4,331	3.63	1.77
25–29	5,365	4.53	1.99
30–34	7,797	6.83	2.80
35–39	11,881	11.42	4.41
40–44	18,041	18.43	7.61
45–49	26,005	28.29	11.79
**Stroke types**			
Hemorrhagic (I60-I62)	59,448	6.88	8.84
Ischemic (I63)	18,675	1.80	3.77
Total	78,123	8.68	12.61
Demographic and clinical characteristics	Deaths (n)	Annual mortality **Southeast**	Annual mortality **South**
**Gender**			
Male	14,178	4.19	3.01
Female	14,446	4.13	3.13
**Age range** (years)			
10–14	309	0.36	0.67
15–19	532	0.61	0.78
20–24	787	0.90	1.32
25–29	1,336	1.54	2.56
30–34	2,416	2.82	3.40
35–39	4,263	5.42	7.71
40–44	7,542	10.13	11.67
45–49	11,439	16.23	17.71
**Stroke types**			
Hemorrhagic (I60-I62)	26,548	3.87	2.81
Ischemic (I63)	2,077	0.29	0.26
Total	28,625	4.16	3.07

Source: Hospital Information System (SIH / SUS) and Mortality Information System (SIM). Data from the Informatics Department of the Unified Health System (DATASUS—https://datasus.saude.gov.br/). Ministry of Health. Brazil.

**Fig 1:**
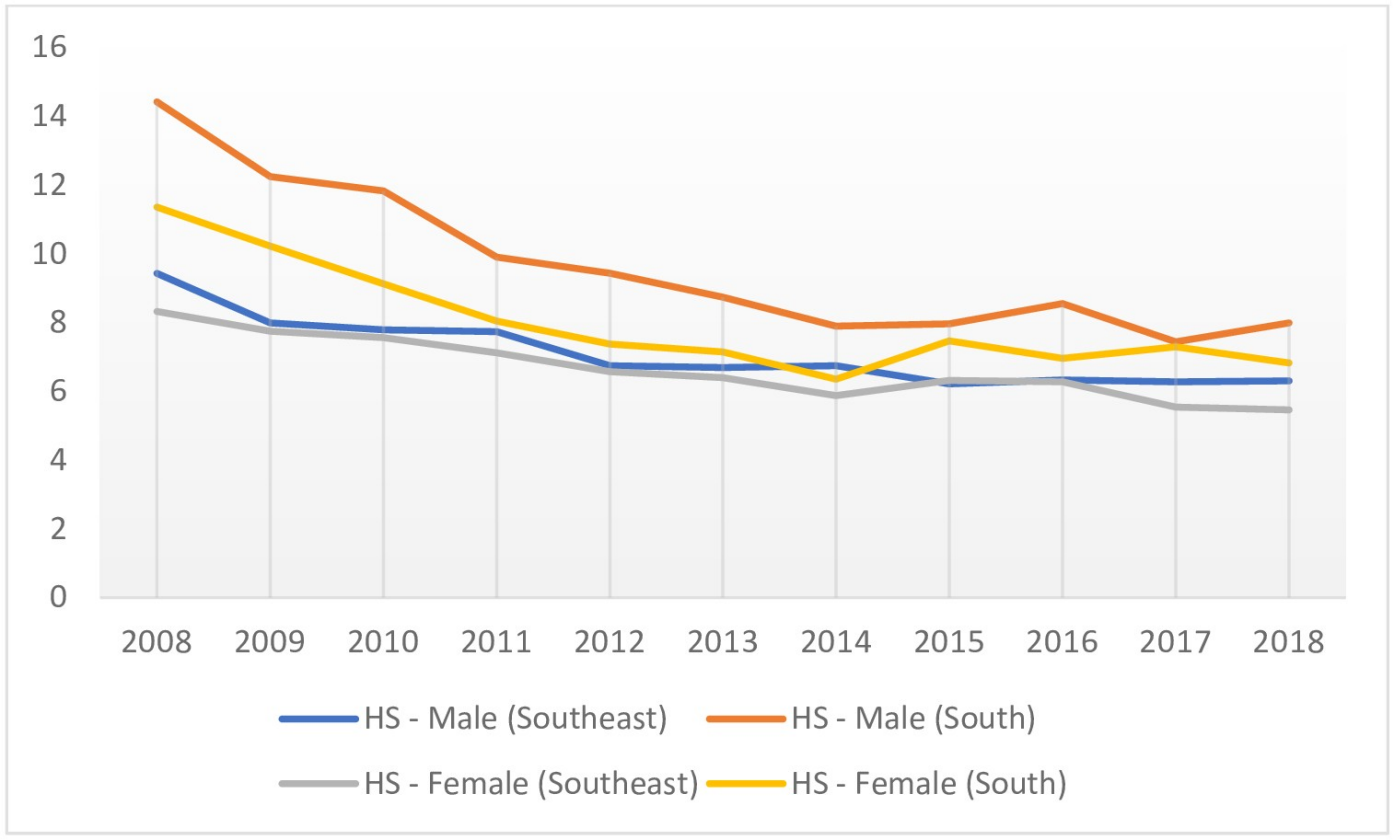
Temporal trend of the incidence of hospitalizations (x100,000 inhabitants) due to haemorragic stroke, stratified by gender in developed regions of Brazil, 2008–2018. HS—Hemorrhagic Stroke; x-axis: years; y-axis: incidence of hospitalization rates.

Incidence of hospitalizations trends revealed a reduction of -4.37% (CI: 95% [-6.24: -2.47]; p = 0.001) due to HS, and a flat tendency for IS (p > 0.005). Hospitalization incidences due to HS decreased -4.43% (CI: 95% [-6.55: -2.27]; p = 0.001) for men, and -4.20% (CI: 95% [-5.82: -2.55]; p < 0.001) for women. For IS, the rates remained unchanged for both gender assessments, and men and women in separate assessments (Table 2, Fig 1).

**Table 2:** Prais-Winsten regression estimates for standardized hospitalization rates due to stroke, stratified by gender in young adults, residents of developed regions of Brazil 2008–2018.

Stroke Standardized Rate	Prais-Winsten Regression			
	β (CI 95%)	*p* Value	APC (CI 95%)	Trend
Developed regions*				
HS	-0.02 (-0.03: -0.01)	0.001	-4.37 (-6.24: -2.47)	Decreasing
HS—Male	-0.02 (-0.03: -0.01)	0.001	-4.43 (-6.55: -2.27)	Decreasing
HS—Female	-0.02 (-0.03: -0.01)	< 0.001	-4.20 (-5.82: -2.55)	Decreasing
IS	0.01 (-0.01: 0.02)	0.353	1.69 (-2.18: 5.72)	Flat
IS—Male	0.00 (-0.01: 0.02)	0.903	0.20 (-3.31: 3.83)	Flat
IS—Female	0.01 (-0.01: 0.03)	0.140	3.07 (-1.20: 7.52)	Flat
**Southeast**				
HS	-0.02 (-0.02: -0.01)	< 0.001	-3.84 (-5.10: -2.57)	Decreasing
HS—Male	-0.02 (-0.02: -0.01)	0.001	-3.71 (-5.39: -1.99)	Decreasing
HS—Female	-0.02 (-0.02: -0.01)	< 0.001	-3.91 (-4.93: -2.89)	Decreasing
IS	0.01 (-0.01: 0.03)	0.220	2.64 (-1.84: 7.32)	Flat
IS—Male	0.00 (-0.01: 0.02)	0.520	1.15 (-2.68: 5.14)	Flat
IS—Female	0.02 (-0.01: 0.04)	0.129	3.86 (-1.34: 9.34)	Flat
**South**				
HS	-0.02 (-0.04: -0.01)	0.003	-5.53 (-8.55: -2.41)	Decreasing
HS—Male	-0.03 (-0.04: -0.01)	0.001	-5.75 (-8.45: -2.97)	Decreasing
HS—Female	-0.02 (-0.04: -0.01)	0.012	-4.75 (-8.04: -1.34)	Decreasing
IS	0.00 (-0.01: 0.02)	0.749	0.48 (-2.77: 3.84)	Flat
IS—Male	0.00 (-0.02: 0.01)	0.660	-0.66 (-3.90: 2.68)	Flat
IS—Female	0.01 (0.00: 0.02)	0.152	2.31 (-1.01: 5.74)	Flat

*Developed regions: assessment of Southeast and South combined.

β—regression coefficient; 95% CI—95% confidence interval; APC: Annual Percent Change (%). Source: SUS Hospital Information System (SIH / SUS). Data from the Informatics Department of the Unified Health System (DATASUS—https://datasus.saude.gov.br/). Ministry of Health. Brazil.

**Fig 2:**
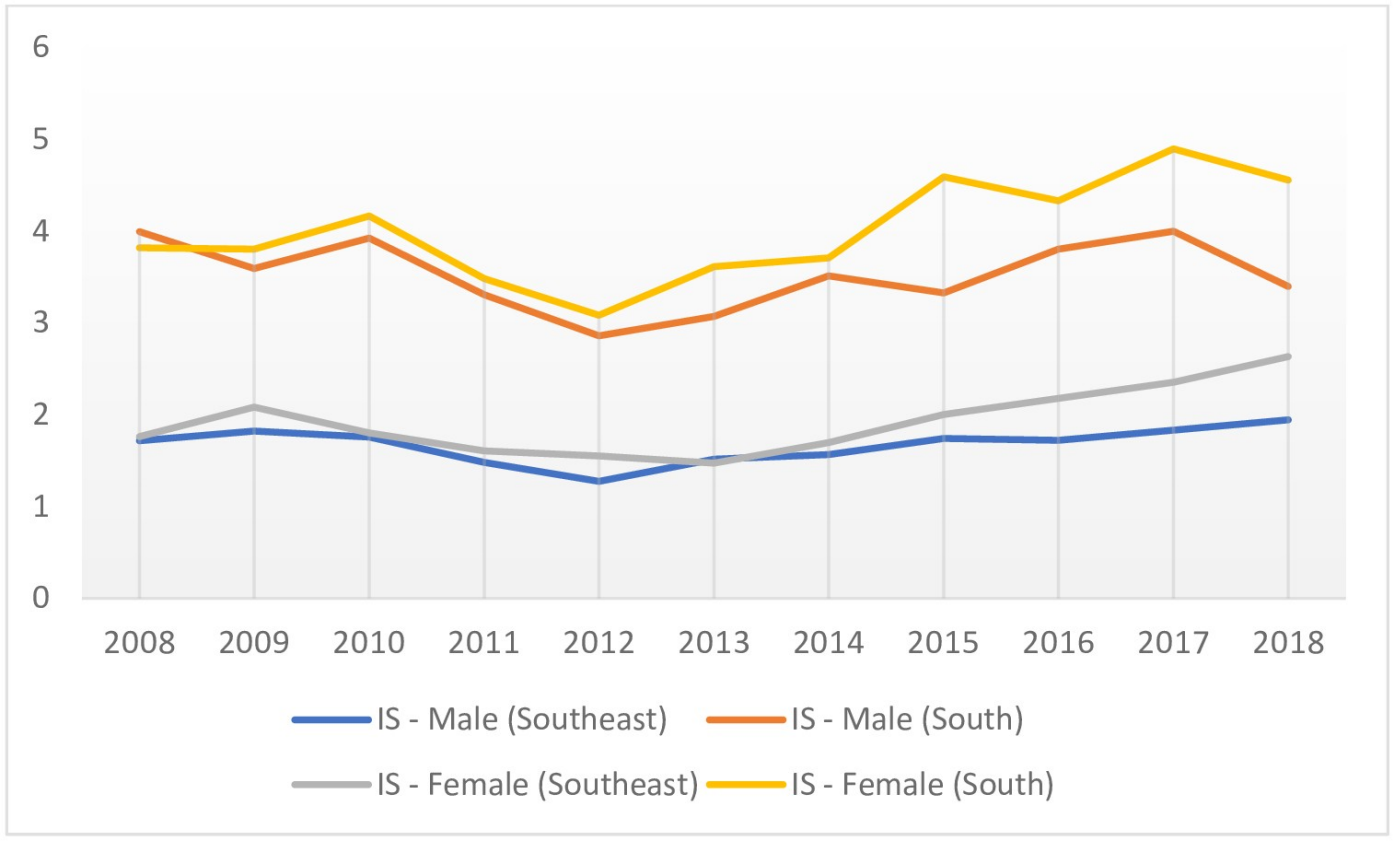
Temporal trend of the incidence of hospitalization (x100,000 inhabitants) due to ischemic stroke, stratified by gender in developed regions of Brazil, 2008–2018. IS—Ischemic Stroke; x-axis: years; y-axis: incidence of hospitalization rates.

However, while the IS hospitalization incidences were flat during the study period (2008–2018), in a recent period (2011–2018), an increase in IS rates was observed. For this time frame (2011–2018), the IS incidence of hospitalization in men in Southeast Brazil increased by 5.4% (CI: 95% [3.7; 7.1], p < 0.001), and in South Brazil by 3.1% (CI: 95% [0.0; 6.3], p = 0.049) (Fig 2). For women, the IS incidence of hospitalization increased by 8.0% in Southeast Brazil (CI: 95% [3.7; 12.4], p = 0.004), and by 6.5% in South Brazil (CI: 95% [3.9; 9, 0], p = 0.001) (Fig 2).

In the Southeast, HS hospitalization incidence rates declined by -3.84% (CI: 95% [-5.10: -2.57]; p < 0.001), when assessing males and females combined. Amongst men, the reduction was -3.71% (CI: 95% [-5.39: -1.99]; p = 0.001), and amongst women, it was -3.91% (CI: 95% [-4.93: -2.89]; p < 0.001) (Table 2; Fig 1). The IS hospitalization incidence rate remained unchanged, for both gender assessment and for gender stratification (i.e. men and women separately) (Table 2, Fig 2).

In the South, HS hospitalization incidence rates declined by -5.53% (CI: 95% [-8.55: -2.41]; p = 0.003), when performed both genders paired. Amongst men, the reduction was -5.75% (CI: 95% [-8.45: -2.97]; p = 0.001), and among women, it was -4.75% (CI: 95% [-8.04: -1.34]; p = 0.012) (Table 2, Fig 2). The IS hospitalization incidence rate remained unchanged, for both gender assessments and for gender stratification (Table 2, Fig 2).

When stratifying by state, the hospitalization incidence rate due to HS declined in all Southeast states, except Espírito Santo, where it remained unchanged (p > 0.005). In São Paulo, the incidence of hospitalizations rate declined by—2.65% (CI: 95% [-3.86: -1.43]; p = 0.001), in Rio de Janeiro by -3.27% (CI: 95% [-3, 83: -2.70]; p <0.001) and in Minas Gerais by -6.96% (CI: 95% [-10.08: -3.72]; p = 0.001) (Table 3).

**Table 3:** Prais-Winsten regression estimates concerning the incidence of hospitalizations rates by type of stroke in young adults, according to states of developed regions of Brazil 2008–2018.

	HEMORRHAGIC (I60-I62)				ISCHEMIC (I63)			
	Prais-Winsten Regression							
	β (CI 95%)	*p* Value	APC (CI 95%)	Trend	β (CI 95%)	*p* Value	APC (CI 95%)	Trend
**Southeast**								
São Paulo	-0.01 (-0.01: -0.01)	0.001	-2.65 (-3.86: -1.43)	Decreasing	-0.02 (-0.01: -0.01)	0.551	1.02 (-2.67: 4.85)	Flat
Rio de Janeiro	-0.01 (-0.01: -0.01)	< 0.001	-3.27 (-3.83: -2.70)	Decreasing	0.02 (-0.01: 0.06)	0.209	5.01 (-3.22: 13.95)	Flat
Minas Gerais	-0.03 (-0.02: -0.02)	0.001	-6.96 (-10.08: -3.72)	Decreasing	0.01 (-0.04: 0.06)	0.586	2.89 (-8.21: 15.33)	Flat
Espírito Santo	-0.02 (0.01: 0.01)	0.230	-3.81 (-10.15: 2.98)	Flat	0.10 (0.02: 0.17)	0.018	24.93 (4.97: 48.70)	Increasing
**South**								
Rio Grande do Sul	-0.02 (-0.04: -0.01)	0.016	-5.54 (-9.59: -1.32)	Decreasing	0.00 (-0.02: 0.01)	0.318	-1.11 (-3.44: 1.28)	Flat
Paraná	-0.03 (-0.04: -0.01)	0.001	-5.61 (-8.15: -3.01)	Decreasing	0.02 (0.00: 0.05)	0.052	5.77 (-0.06: 11.95)	Flat
Santa Catarina	-0.02 (-0.03: -0.01)	< 0.001	-4.40 (-6.19: -2.58)	Decreasing	0.01 (0.00: 0.03)	0.139	2.81 (-1.09: 6.87)	Flat

β—regression coefficient; 95% CI—95% confidence interval; APC: Annual Percent Change (%). Source: SUS Hospital Information System (SIH / SUS). Data from the Informatics Department of the Unified Health System (DATASUS—https://datasus.saude.gov.br/). Ministry of Health. Brazil.

In the South, HS hospitalization incidences decreased in all states: Rio Grande do Sul by -5.54% (CI: 95% [-9.59: -1.32]; p = 0.016); Paraná by -5.61% (CI: 95% [-8.15: -3.01]; p = 0.001) and Santa Catarina by -4.40 (CI: 95% [-6.19: -2.58]; p < 0.001) (Table 3).

In terms of IS hospitalization incidences, rates remained unchanged in all states (p > 0.005), except Espírito Santo, where an IS increase of 24.93% (CI: 95% [4.97: 48.70]; p = 0.018) was observed (Table 3).

### Mortality

In total, 28,625 stroke deaths were recorded, of which 26,548 (92.7%) were due to HS. Death rates in the Southeast were higher than those in the South during the years of study, and all age groups. In terms of gender stratification, the annual mortality (x 100,000 persons) for men was 4.19 in the Southeast, and 3.01 in the South. The annual mortality (x 100,000 persons) for women was 4.13 in the Southeast, and 3.13 in the South (Table 1).

**Fig 3:**
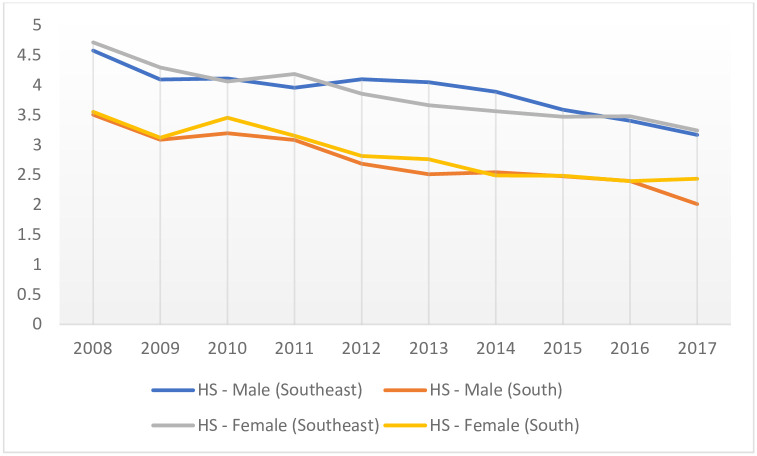
Temporal trend of the mortality (x100,000 inhabitants) due to hemorrhagic stroke, stratified by gender of young adults, residents of developed regions of Brazil, 2008–2017. HS—Hemorrhagic Stroke; x-axis: years; y-axis: mortality rates.

**Fig 4:**
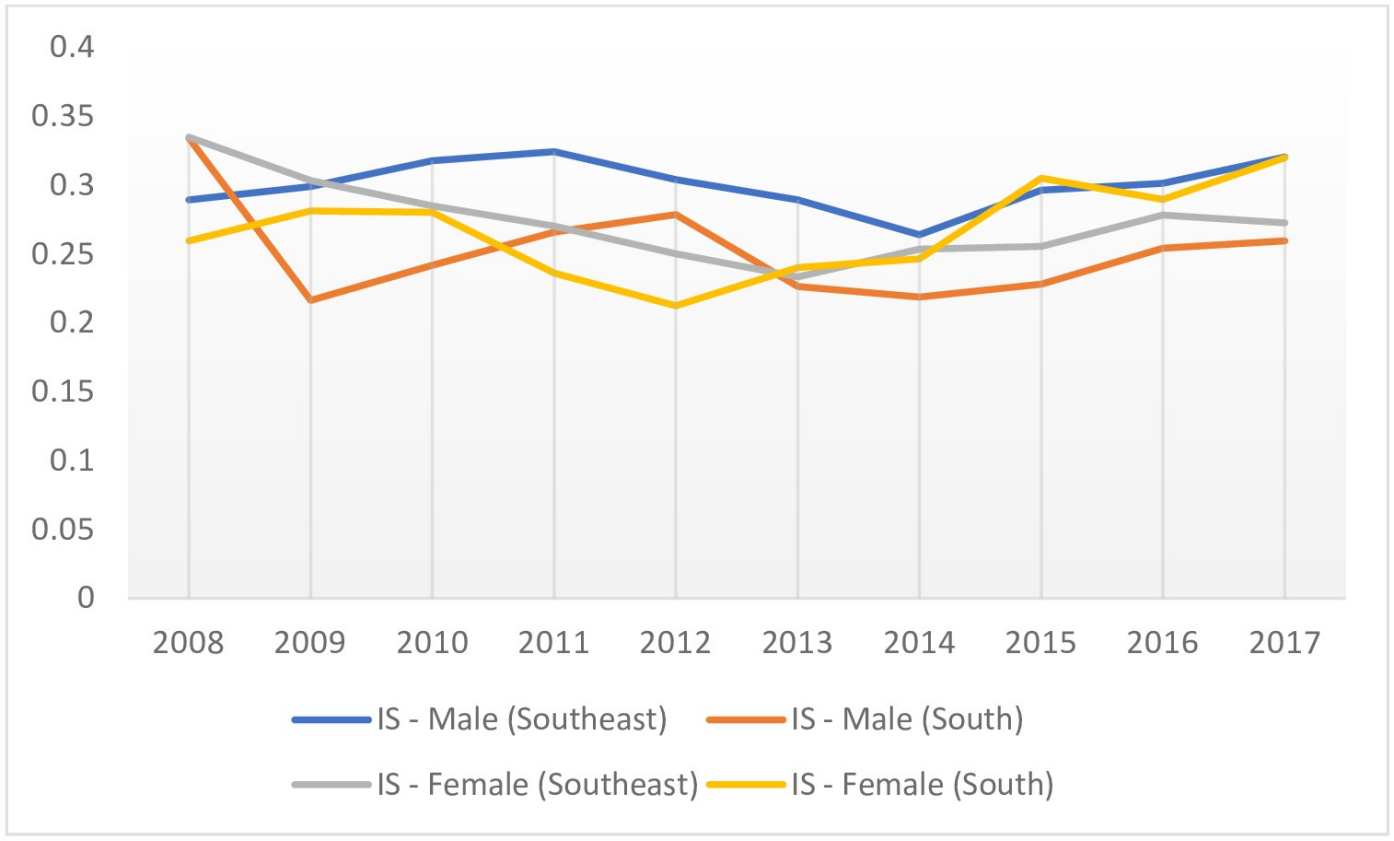
Temporal trend of the mortality (x100,000 inhabitants) due to ischemic stroke, stratified by gender of young adults, residents of developed regions of Brazil, 2008–2017. IS—Ischemic Stroke; x-axis: years; y-axis: mortality rates.

Statistical analyzes revealed a reduction of -3.48% (CI: 95% [-3.88: -3.09]; p < 0.001) in HS mortality rates in developed regions of Brazil, when performing both genders (i. e., male and female) assessment. When stratifying according to stroke type and gender, HS mortality in women declined by -3.83% (CI: 95% [-5.07: -2.57]; p < 0.001), whereas the rate was flat for men (Table 4, Fig 3). For IS, assessing male and female combined, a decreasing trend of -3.84% was determined (CI: 95% [-4.52: -3.16]; p < 0.001). However, by gender stratification (i. e., male and female separately), rates remained unchanged for both men and women (Table 4, Fig 4).

**Table 4:** Prais-Winsten regression estimates for standardized mortality rates due to stroke, stratified by sex in young adults, residents of developed regions of Brazil 2008–2017.

Stroke Standardized Rate	Prais-Winsten Regression			
	β (CI 95%)	*p* Value	APC (CI 95%)	Trend
**Developed regions**				
HS (joint assessment)	-0.02 (-0.02: -0.01)	<0.001	-3.48 (-3.88: -3.09)	Decreasing
HS—Male	-0.01 (-0.02: 0.01)	0.353	-1.37 (-4.49: 1.86)	Flat
HS—Female	-0.02 (-0.02: -0.01)	<0.001	-3.83 (-5.07: -2.57)	Decreasing
IS (joint assessment)	-0.02 (-0.02: -0.01)	<0.001	-3.84 (-4.52: -3.16)	Decreasing
IS—Male	0.00 (-0.02: 0.01)	0.510	-0.96 (-4.12: 2.29)	Flat
IS—Female	-0.01 (-0.03: 0.01)	0.338	-1.91 (-6.1: 2.47)	Flat
**Southeast**				
HS (joint assessment)	-0.01 (-0.02: -0.01)	<0.001	-3.33 (-3.94: -2.72)	Decreasing
HS—Male	-0.02 (-0.02: -0.01)	0.001	-3.56 (-5.10: -1.98)	Decreasing
HS—Female	-0.02 (-0.02: -0.01)	<0.001	-3.67 (-4.34: -2.99)	Decreasing
IS (joint assessment)	-0.01 (-0.02: 0.01)	0.275	-1.39 (-4.07: 1.36)	Flat
IS—Male	0.00 (-0.01: 0.01)	0.759	-0.38 (-3.12: 2.43)	Flat
IS—Female	-0.01 (-0.03: 0.00)	0.059	-3.11 (-6.26: 0.15)	Flat
**South**				
HS (joint assessment)	-0.02 (-0.03: -0.02)	<0.001	-5.12 (-5.97: -4.27)	Decreasing
HS—Male	-0.02 (-0.03: -0.02)	<0.001	-5.12 (-6.29: -3.93)	Decreasing
HS—Female	-0.02 (-0.03: -0.01)	<0.001	-4.54 (-5.68: -3.39)	Decreasing
IS (joint assessment)	-0.01 (-0.03: 0.01)	0.441	-1.59 (-5.97: 3.00)	Flat
IS—Male	-0.01 (-0.05: 0.02)	0.375	-3.05 (-10.16: 4.61)	Flat
IS—Female	0.00 (-0.02: 0.03)	0.861	0.45 (-5.11: 6.33)	Flat

*Developed regions: assessment of Southeast and South combined.

β—regression coefficient; 95% CI—95% confidence interval; APC: Annual Percent Change (%). Source: SUS Hospital Information System (SIH / SUS). Data from the Informatics Department of the Unified Health System (DATASUS—https://datasus.saude.gov.br/). Ministry of Health. Brazil.

When stratifying by region, HS mortality rates declined by -3.33% (CI 95% [-3.94: -2.72]; p < 0.001) in the Southeast, when evaluating both genders combined. For men, the reduction was -3.56% (CI: 95% [-5.10: -3.16]; p = 0.001), and for women, it was -3.67% (CI: 95% [-4, 34: -2.99]; p < 0.001) (Table 4, Fig 3). IS rates remained unchanged, for both gender assessments and gender stratification (Table 4, Fig 4).

In the South, HS mortality rates declined by -5.12% (CI: 95% [-5.97: -4.27]; p < 0.001) evaluating male and female paired. For men, the reduction was also -5.12% (CI: 95% [-6.29: -3.93]; p < 0.001), and for women it was -4.54% (CI: 95% [-5.68: -3.39]; p < 0.001) (Table 4, Fig 3). IS rates remained unchanged, for both gender combined assessments and gender stratification (Table 4, Fig 4).

Stratification by states showed statistically significant declining HS mortality rates in all Southeast and South states (p < 0.05). In São Paulo, the rate reduction was -3.17% (CI: 95% [-3.77: -2.57]; p < 0.001); in Rio de Janeiro it was -2.43% (CI: 95% [-3.36: -1.48]; p < 0.001); in Minas Gerais it was -4.69% (CI: 95% [-6.17: -3.17]; p < 0.001) and in Espírito Santo it was -4.83% (CI: 95% [-6.36: -3.27]; p <0.001).

In the South, HS mortality rates declined by -4.50% (CI: 95% [-5.31: -3.68]; p <0.001) in Rio Grande do Sul, in Paraná it was -5.15% (CI: 95% [-6.50: -3.78]; p <0.001), and in Santa Catarina it was -4.19% (CI: 95% [-5.92: -2.43]; p = 0.001) (Table 5).

**Table 5:** Prais-Winsten regression estimates concerning mortality rates by type of stroke in young adults, according to states of developed regions of Brazil 2008–2017.

	HEMORRHAGIC (I60-I62)				ISCHEMIC (I63)			
	Prais-Winsten Regression							
	β (CI 95%)	*p* Value	APC (CI 95%)	Trend	β (CI 95%)	*p* Value	APC (CI 95%)	Trend
**Southeast**								
São Paulo	-0.01 (-0.02: -0.01)	< 0.001	-3.17 (-3.77: -2.57)	Decreasing	0.00 (-0.01: 0.02)	0.486	1.01 (-2.14: 4.25)	Flat
Rio de Janeiro	-0.01 (-0.01: -0.01)	< 0.001	-2.43 (-3.36: -1.48)	Decreasing	0.01 (-0.01: 0.04)	0.280	3.25 (-3.13: 10.06)	Flat
Minas Gerais	-0.02 (-0.03: -0.01)	< 0.001	-4.69 (-6.17: -3.17)	Decreasing	-0.07 (-0.09: -0.05)	< 0.001	-14.75 (-18.97: -10.30)	Decreasing
Espírito Santo	-0.02 (-0.03: -0.01)	< 0.001	-4.83 (-6.36: -3.27)	Decreasing	-0.02 (-0.09: 0.04)	0.400	-5.45 (-18.47: 9.64)	Flat
**South**								
Rio Grande do Sul	-0.02 (-0.02: -0.02)	< 0.001	-4.50 (-5.31: -3.68)	Decreasing	0.01 (-0.02: 0.04)	0.507	2.07 (-4.66: 9.28)	Flat
Paraná	-0.02 (-0.03: -0.02)	< 0.001	-5.15 (-6.50: -3.78)	Decreasing	-0.02 (-0.05: 0.02)	0.323	-3.81 (-11.65: 4.72)	Flat
Santa Catarina	-0.02 (-0.03: -0.01)	0.001	-4.19 (-5.92: -2.43)	Decreasing	0.03 (-0.05: 0.11)	0.390	7.25 (-10.21: 28.10)	Flat

β—regression coefficient; 95% CI—95% confidence interval; APC: Annual Percent Change (%). Source: SUS Hospital Information System (SIH / SUS). Data from the Informatics Department of the Unified Health System (DATASUS—https://datasus.saude.gov.br/). Ministry of Health. Brazil.

In terms of IS rates, mortality declined only in Minas Gerais, -14.75% (CI: 95% [-18.97: -10.30]; p < 0.001). In other Southeast states and in all South states, IS mortality rates remained unchanged (p > 0.05) (Table 5).

## Discussion

During the study period, Hemorrhagic Stroke (HS) hospitalization incidences declined, whereas Ischemic Stroke (IS) incidence of hospitalizations remained unchanged in developed regions of Brazil. However, in a recent period (2011–2018), an increase in IS hospitalizations incidence rates were observed in men and women. In Espírito Santo, the incidence of hospitalization enhanced between 2008–2018 over than Southeast region in the recent period. Mortality rates due to HS decreased and remained flat for IS in the Southeast and South. Nevertheless, IS mortality rates declined in Minas Gerais.

Our study shows that incidence of hospitalizations and mortality rates for stroke exponentially increased with age, following Ekker et al. findings, whose showed the same trend in a cohort study conducted in the Netherlands regarding individuals aged 18–50 years-old, between 1998–2010 [19]. Considering the physiopathology of the disease, it is an expected scenario since elder individuals are more exposed to risk factors, such as hypertension, dyslipidemia and diabetes [20]. Moreover, in line with this Dutch study and another marked out in Finland (2004–2014), we also found increase in stroke hospitalization incidence rates limited to IS [21]. In Brazil, it was already pointed by Cabral et al. (2017), who observed a 66% increase in IS incidence rate ratios in adults aged < 55 (IRR = 1.66; CI: 95%, 1.09–2.54) [7].

This observation may be attributed to the risk factors in young populations related to the IS, such as overweight, a lack of physical activity and systemic hypertension [22]. A study performed in Greater Cincinnati/Northern Kentucky region attributed an increase in the prevalence of vascular risk factors, including diabetes mellitus, obesity, and illicit drug use in younger people to the increase in incidence of IS [23]. Regarding specifically the drug use, the increasing in IS rates in Brazil are possibly related to the higher prevalence of drug use than other Latin American countries [24]. The Southeast, in particular, has the highest prevalence of cocaine use: 5.7% of the population uses cocaine at least once in their lifetime [24]. Additionally, the increasing in incidence IS rates is not unexpected in low-income and middle-income countries, where awareness of risk factors is not accessible for the whole population, levels of primary and follow-up health care are low, and basic drugs and equipment for the prevention and treatment of stroke are scarce [25].

Despite IS hospitalization increase, no increasing rates related to mortality were reported. This may be attributed to the higher levels of health resource access and the better socioeconomic conditions in the South and Southeast, when compared to the rest of the country [26, 27]. São Paulo has public health system hires private primary care services mainly to increase the number of health professionals, in order to substitute outdated processes (manuals, non-standardized systems) and the lack of specialized human resources [28]. Moreover, the number of private health plans in developed regions of Brazil has increased in the past years, particularly in São Paulo [28], which contrasts to lower socioeconomic groups, more often covered by the public health system. It demonstrates that access to the private system in Brazil is a key to the prognosis of the disease [29], what could explain why mortality rates for IS did not increase in developed regions in Brazil.

Brazil is divided into five regions. The Southeast has the highest population, followed by the South, Northeast, Midwest and Northern regions [30]. Different from South and Southeast, Northeast has lower socioeconomic conditions, containing approximately 61% of the 1,431 Brazilian municipalities with a low human development index [30]. It was reflected in the increase of 20.66 (CI: 95% 20.65; 20.66) to 24.90 (CI: 95% 24.89; 24.90) (β = 1.14, p = 0.079) observed for hospitalization incidences in young adults living in the Northeast, between 2012–2014 [1]. Adami et al. findings contrast with ours, since we found an overall reduction in HS hospitalization incidence and mortality rates in the Southeast and South (developed regions). These contrasting results supports the assertion indicating that socioeconomic regions differences may influence in the prognosis of the disease.

In our study, stratification by state showed a significant increase in IS hospitalization incidences in Espírito Santo, and a reduction in IS mortality in Minas Gerais. Moreover, disease type and trend rates in states varied significantly, suggesting that even in developed regions, socioeconomic differences may have influenced in stroke hospitalization incidence and mortality in younger populations. It was interesting to note that the only state where HS hospitalization rates remained unchanged (Espírito Santo), was the same state with significantly increasing IS rates. This phenomenon was also observed in the literature: in 2019, a chronic kidney disease (CKD) study conducted in Espírito Santo, revealed increasing hospitalization rates in 20–49-year-old populations [31]. Although stroke and CKD are distinctly different diseases, our findings and this study [31] demonstrates that young people in Espírito Santo may be at higher risk for developing chronic diseases.

Therefore, the decline in HS hospitalization incidence rates observed in this study diverged with a population-based study, conducted in Joinville, Brazil, between 2005 and 2015, evaluating Brazilian young adults, which showed a flat trend [7]. Despite of the differences in this study design from ours, results are comparable since we have standardized rates (x 100.000 persons), evaluating the same outcome in a similar age (young adults). The reduction in HS hospitalization incidence and mortality in young populations may be attributed to the Family Health Strategy (FHS), which since 1994 has reorganized primary health services to ensure universal health access, thereby improving health-related education and raising health promotion indices [32]. Cabral et al. described the decrease in smoking was the most remarkable feature of premorbid risk profile for stroke in young adults of Brazil [7]. Krishnamurthi et al. (2014) attributed the reduction in mortality rates due to HS because of the advances in diagnosis of stroke type, and more targeted health care in some developing regions in low-income to middle-income countries in the past 2 decades, particularly because low-income to middle-income countries might be more heterogeneous than high-income countries [25].

The stroke incidence rate found in our study in young individuals (24%), in relation to the total population, contrasts with studies by Marini *et al*. (2011) and Maaijwee *et al*. (2014), who determined stroke incidences in the young population ranging from 5%–10% [5, 10]. Although the highest proportion of HS was observed in young patients (40–55%) when compared to the general population (15–20%), HS is still less common than IS [22, 24]. Our study showed a huge percentual of HS occurring in young adults: HS cases corresponded to 76.1% of total, and HS deaths 92.8%. Compared to high-income countries, these percentages are alarming, since the mortality in these countries are substantially lower. In individuals < 20 years living in low-income countries, mortality is 6 times greater than those who are living in high-income countries (p < 0.001). Regarding adults aged ≥ 20–64 years, mortality is 1.8 times higher in low-income nations (p < 0.001) [25]. This phenomenon could be explained by the high prevalence of hypertension in Brazil: 30,5% were reported to be diagnosed with the disease. In relation to other Latin countries, Brazil is the fourth nation with the highest prevalence of hypertension [33].

Few studies have examined the cause of HS, however different etiologies may be influenced by ethnicity. One study suggested that hypertension was the most commonetiology, accounting for 64% of HS in people < 45 years, however 88% of the study population was black [34]. A Chinese study recently evaluated 401 patients < 45 years old for etiologies and risk factors for intracerebral hemorrhage and suggested that systemic arterial hypertension was the most common etiology in 226 patients (56.4%). 41 (10.2%) patients had congenital cerebrovascular disease, and 27 (0.25%) patients had other etiologies, such as moyamoya disease, cerebral venous thrombosis, drug abuse, hemorrhagic tumors or autoimmune causes [35]. Regardless of the risk of cerebrovascular malformation-related bleeding was higher in the 20–29-year-old group, the risk of hypertension-related bleeding increased with age, despite the same underlying risk factors of smoking and alcohol [30]. The resultant increase in the prevalence of diabetes, with increases in smoking rates and increasingly sedentary lifestyles, has contributed to the increased atherosclerotic disease [36]. During different phases of epidemiological transition, increased incidence of hemorrhagic stroke is expected, particularly in low-income and middle-income countries, because hypertension is the dominant risk factor for this stroke type [25].

Brazil has the second highest age-adjusted loss of life in all South American countries, and one of the highest risk rates for premature stoke death in the world [37]. These results are concerning since stroke in the young population can highly impact in their quality of life, as subsequently the economy, as the consequences can disable the youngers to work in their most productive years of life [4, 14]. To mitigate these consequences, developed regions in Brazil had experienced an improving in health resources in the past decades, although the lack of evidence-based management guidelines is challenging for these unusual research-based etiologies that have been completed to date [21, 38]. In addition to studies evaluating stroke occurrence in specific population subgroups, more studies are required to monitor trends in other risk factors, such as tobacco use, drug distribution, quality of health care and socioeconomic conditions.

Our study did not consider I64 for trend analysis because of the large proportion of this stroke type. Regarding only developed regions and young adults (10–49 years old), I64 hospitalizations represented just over half of all stroke types, consistently increasing from 48% in 2008, to 62% in 2018. On average, the proportion for the younger was 58% between 2008–2018, whereas it was 74% among all individuals. Although the average for the younger is lower compared to the total, it is still high, what could mask the results for I63, since authors tend to regard both codes as ischemia when analyzing all individuals [39]. In our population, between 2008–2018, mortality for I64 decreased from 28% to 21%. The proportion of deaths due to I64 is significantly lower than for hospitalizations. Concerning the proportion of deaths due to stroke out of hospital in the young population, rates remained flat between 1996–2015 in São Paulo, the bigger state of the developed regions [38]. Thus, deaths occurred out of the hospital did not interfere with our analysis.

There are several limitations in our study. Two are related to the inherit restrictions of the databases used. With respect to SIH/SUS, which did not distinguish first hospitalizations between readmissions, the database could overestimate rates, as long as it does not exclude patients admitted due to stroke in previous years. This database also does not allow to distinguish I60, I61 and I62, and consider all these codes as hemorrhagic stroke [1], what could hide differences between codes. We did not use I64 code, which could represent a limitation because of the large proportion of this stroke type in our population. Furthermore, we emphasize that ecological retrospective studies using secondary database does not allow access to individual’s information. This study design considers population group as a whole and do not consider particular conditions of individuals.

This is the first epidemiological study using official stroke data for hospitalizations and death rates in young people to stratify by gender and type of stroke, for the two most developed regions of Brazil, over a period of 11 consecutive years. These data highlight the importance of these epidemiological studies, as they contribute to increased understandings between the environment, the individual and society, as well as significantly impact on changes in health systems and technological development, thereby potentially limiting disease burdens on populations [40].

## Conclusions

We identified a decline in HS hospitalization incidences, and a flat tendency for IS hospitalizations in developed regions of Brazil. However, between 2011 and 2018, we observed an increase in IS rates in men and women living in both developed regions. Mortality rates due to HS and IS were decreased between 2008 and 2018 in the Southeast and South. Significant variations in the magnitude and trend of rates according to disease types in Brazilian states were observed, which suggests that, even in developed regions, differences in socioeconomic factors may influence stroke epidemiology in young adults.
